# Evolution and expression analyses of the MADS-box gene family in *Brassica napus*

**DOI:** 10.1371/journal.pone.0200762

**Published:** 2018-07-19

**Authors:** Yunwen Wu, Yunzhuo Ke, Jing Wen, Pengcheng Guo, Feng Ran, Mangmang Wang, Mingming Liu, Pengfeng Li, Jiana Li, Hai Du

**Affiliations:** 1 College of Agronomy and Biotechnology, Chongqing Engineering Research Center for Rapeseed, Southwest University, Chongqing, China; 2 Academy of Agricultural Sciences, Southwest University, Chongqing, China; Huazhong University of Science and Technology, CHINA

## Abstract

MADS-box transcription factors are important for plant growth and development, and hundreds of MADS-box genes have been functionally characterized in plants. However, less is known about the functions of these genes in the economically important allopolyploid oil crop, *Brassica napus*. We identified 307 potential MADS-box genes (*BnMADSs*) in the *B*. *napus* genome and categorized them into type I (M_α_, M_β_, and M_γ_) and type II (MADS DNA-binding domain, intervening domain, keratin-like domain, and C-terminal domain [MIKC]^c^ and MIKC*) based on phylogeny, protein motif structure, and exon-intron organization. We identified one conserved intron pattern in the MADS-box domain and seven conserved intron patterns in the K-box domain of the MIKC^c^ genes that were previously ignored and may be associated with function. Chromosome distribution and synteny analysis revealed that hybridization between *Brassica rapa* and *Brassica oleracea*, segmental duplication, and homologous exchange (HE) in *B*. *napus* were the main *BnMADSs* expansion mechanisms. Promoter cis-element analyses indicated that *BnMADSs* may respond to various stressors (drought, heat, hormones) and light. Expression analyses showed that homologous genes in a given subfamily or sister pair are highly conserved, indicating widespread functional conservation and redundancy. Analyses of *BnMADSs* provide a basis for understanding their functional roles in plant development.

## Introduction

The MADS-box gene family includes important transcription factors that are relevant across Eukarya, including fungi, animals, and plants [1]. This gene family commonly possesses a highly conserved N-terminal DNA binding domain (MADS-box), a moderately conserved coiled-coil domain for protein reciprocity (K-box), a lightly conserved domain (I domain) between the MADS-box and K-box, and a non-conserved C-terminal. The MADS-box is a DNA binding domain of approximately 58 amino acids that binds DNA at consensus recognition sequences known as CArG boxes [CC(A/T)6GG][2, 3]. The I terminal is less conserved and contributes to the specification of dimerization. The K domain is characterized by a coiled-coil structure that facilitates the dimerization of MADS-box proteins [4, 5]. Evolution of motifs in different C-terminals results in different functions for MADS-box proteins [4, 6].

The MADS-box gene family is divided into two main lineages, type-I and type-II, that originated from a duplication of ancestor genes during the divergence of plants and animals [7]. Type-I genes were further divided into an animal serum-response factor (SRF) type and plant type-I, while type-II genes were divided into fungi myocyte enhancer factor 2 (MEF2) type and plant type-II[7]. A major difference between type-I and type-II genes is that type-II genes possess the K-box domain. Genes in the type-II lineage are also termed MADS DNA-binding domain, intervening domain, keratin-like domain, and C-terminal domain (MIKC) MADS-box genes, attributable to their four domains, including the MADS-box domain, I domain, K-box domain, and C terminal [8, 9]. MIKC-type genes can be further divided into MIKC^c^ and MIKC* clades, both of which were identified in a common ancestor of moss and vascular plants, suggesting they are ancestral gene types [10].

The MADS-box gene family is known to contain flower organ identity genes and participate in homologous transformation during floral ontogeny. Floral organ identity genes are subdivided into A, B, C, D, and E classes (ABCDE model), providing different homeotic functions with different combinations, including A sepal, A+B petal, B+C stamen, C carpel, D ovary formation and ovule development, E formation of floral organ in four rounds, and B+C+E male flowers[11–15]. Moreover, the MADS-box gene family also contains flowering-time genes and fruit dehiscence-related genes [16–18]. In addition, MADSs play regulatory roles during the vegetative stage, including embryo, root, and leaf development [16, 19–22]. MADSs also have been identified in gymnosperms and mosses without flowers or fruits, proving they are not limited to flower and fruit development [10, 23–27].

To date, studies regarding the MADS-box gene family have been performed at the genome level in many plant species, including *Arabidopsis* and *Brassica rapa* [28, 29]. *Brassica napus* is one of the most important oil crops and ornamental plants in China; *B*. *napus* fluoresces, which is considered decorative, while its seeds can be used to produce edible oils. Thus, identification and functional analyses of the MADS-box gene family in *B*. *napus* would contribute to our understanding of the developmental mechanisms associated with flowering, as well as improving floral organ features. For example, silique dehiscence is a serious problem that results in a reduction in seed yield in *B*. *napus* [30], and the molecular mechanisms underlying silique dehiscence are closely related to the ABCDE model. Thus, corresponding analyses in *B*. *napus* could be an alternative way to solve this problem.

In this study, we performed a complete analysis of the MADS-box family of genes in *B*. *napus* (*BnMADSs*) by identifying putative genes, evaluating the division of different subfamilies based on phylogeny, and exploring the protein structure and exon-intron characters. We proved that the evolutionary mechanisms underlying intron patterns remain consistent with those of functional diversification. Chromosome localization combined with gene synteny analyses revealed the expansion of *BnMADSs* in *B*. *napus*. In addition, a cis-acting regulatory element analysis of promoters indicated possible roles for *BnMADSs* in plant development and stress response. Moreover, an expression profile analysis revealed abundant roles for *BnMADSs* in *B*. *napus*.

## Material and methods

### Sequence retrieval

A preliminary search for *B*. *napus* MADS-box proteins in Genoscope (http://www.genoscope.cns.fr/brassicanapus/) was performed using the basic local alignment search tool-protein (BLASTP) with at least one representative sequence of the MADS-box domain for each MADS-box subfamily. We discarded redundant sequences with the same chromosome locus to ensure the candidate genes mapped to unique loci in their respective genomes. We then confirmed the putative non-redundant sequences to ensure that the putative proteins contained MADS-box domains using ExPASy (http://expasy.org/prosite/) [31] and MEGA 7.0 [32] software. Finally, all candidate genes were named according to the chromosome locus.

### Phylogenetic tree construction

Multiple sequence alignment was performed using MADS-box protein sequences of *Arabidopsis* and *B*. *napus* via the multiple alignment fast Fourier transform (MAFFT) software (http://mafft.cbrc.jp/alignment/server/) [33]. The phylogenetic tree was constructed based on alignment of the MADS-box domains using MEGA7.0 [32] and the neighbor-joining (NJ) method, with 1000 iterations for the bootstrap values, p-distance model, and pairwise deletion for gap treatment. Tree files were viewed and edited using FigTree v1.3.1 (http://tree.bio.ed.ac.uk/software/figtree/).

### Chromosome localization

Information regarding chromosome length and gene locations was obtained from the Brassica Database (http://brassicadb.org/brad/index.php) and *B*. *napus* Genome Browser (http://jacob.cea.fr/drf/ifrancoisjacob/Pages/Departements/Genoscope.aspx), respectively. Mapchart software was used to draw a chromosome map of the *BnMADSs*. Locations of the MADS-box genes in *Arabidopsis*, *B*. *rapa*, and *Brassica oleracea* were also determined using the same method.

### Intron/Exon structure analysis

To find the intron and exon distribution and splicing phase in the candidate *BnMADSs*, we compared and viewed the coding DNA sequences (CDS) and DNA sequences of *BnMADSs* using GSDS software (http://gsds.cbi.pku.edu.cn/) [34] and then manually located the intron insertion sites of protein sequences. Information about the MADSs intron insertion sites in other species included in this study were gathered from Phytozome (https://phytozome.jgi.doe.gov/pz/portal.html#).

### Cis-element analysis of promoters

Cis-acting regulatory elements as binding sites of transcription factors connect gene structure and function [35] and play important roles in gene expression. The cis-elements in *BnMADSs* promoters were predicted using PlantCARE (http://bioinformatics.psb.ugent.be/webtools/plantcare/html/).

### Identification of conserved motifs

Full-length protein sequences of candidate *BnMADSs* were analysed using MEME software (http://meme-suite.org/tools/meme) [36], with the following parameters: optimum motif width ≥ 6 and ≤ 150 and maximum number of motifs to identify = 15. In addition, we used the PFAM tool (http://pfam.xfam.org/) to identify whether any remaining motifs matched well-known motifs [37].

### Gene synteny analysis

We used CoGe software (https://genomevolution.org/coge/) to conduct a gene synteny analysis of MADS-box genes in *Arabidopsis*, *B*. *napus*, *B*. *rapa*, and *B*. *oleracea*.

### Expression analysis of *BnMADSs*

The temporal and spatial expression patterns of candidate *BnMADSs* were further analysed using the RNA-seq data of 50 different tissues (including roots, stems, leaves, flowers, seeds, and siliques) from the *B*. *napus* cultivar Zhongshuang 11 (ZS11) at different developmental stages (germination, seedling, budding, initial flowering, and full-bloom stages). We recently created and deposited ZS11 in the BioProject (ID PRJNA358784). *BnMADSs* expression was analysed using Cluster 3.0 software based on fragments per kilobase of exon per million reads mapped (FPKM) with default parameters, and the heatmap was created using Cluster 3.0 [38] and Java Treeview software [39].

## Results

### Identification of MADS-box genes in *B*. *napus*

With the aim of defining the *B*. *napus* MADS-box gene family, we applied BLASTP for genes encoding MADS-box proteins in Genoscope using representative DNA-binding domain sequences of typical MADS-box proteins as queries (S1 Table). After removing redundant sequences, 312 primary putative MADS-box proteins were obtained in *B*. *napus* for this study.

To verify the reliability, we performed a PROSITE profile analysis to ensure all the putative MADS-box protein sequences contained the typical MADS-box domain. Consequently, BnaA08g31660D and BnaA09g05900D were deleted, as they had poor homology with atypical MADS-box proteins; BnaA09g30870D, BnaA02g31770D, and BnaC05g45970D were deleted because of mass deletions, especially at the N-terminal; while BnaC01g34010D, BnaC02g43730D, BnaAnng04820D, and BnaA05g35580D were reserved because the deletion occurred at the C-terminal, which did not impact the subsequent phylogenetic analyses.

Finally, a total of 307 *BnMADSs* with relatively complete open reading frame (ORF) regions were identified in this study, constituting the largest known plant MADS-box gene family to date. The candidate genes were then named according to the chromosomal distribution order (S1 Table).

### Sequence characteristics of BnMADS proteins

To investigate sequence features, we performed a multiple alignment analysis of type-I and type-II BnMADS proteins, respectively. The MADS-box domain of type-I proteins is relatively less conserved and studied than those of type-II proteins; therefore, we manually identified type-I proteins using type-I MADS-box proteins in *Arabidopsis* as a reference [28].

**Fig 1:**
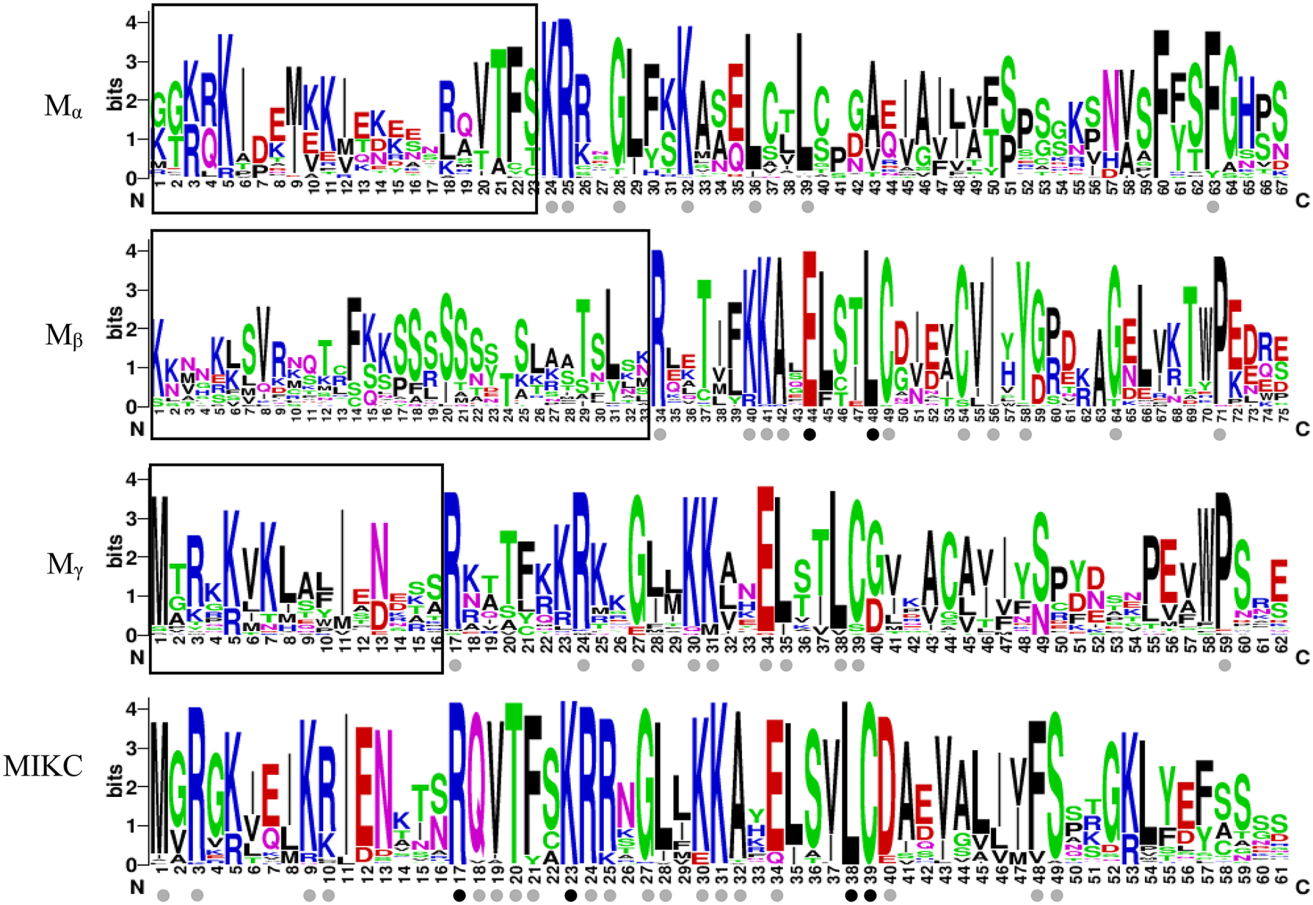
MADS-box domain of MADSs-box genes in the *Brassica napus* (*BnMADSs*) genome. A multiple alignment analysis was performed using the MAFFT program. The sequence logos are based on the alignments of all type I (M_α_, M_β_, and M_γ_) and type II (MIKC) *B*. *napus* MADS-box domains. Bit scores indicate the information content for each position in the sequence. Black and grey dots indicate 100%- and 90%-conserved residues, respectively.

Our results show that MADS-box domains of type-I proteins are quite divergent in length and sequence characteristics (Fig 1). The length of the MADS-box domains in M_α_, M_β_, and M_γ_ were 67aa, 75aa, and 62aa, respectively. In type-I, the N-terminal of the MADS-box domain was relatively more divergent than the C-terminal. In contrast, the MADS-box domains of type-II proteins were highly conserved, and the basic region consisted of 61 basic residues with rare deletions or insertions (approximately 1.6%). The residues Arg-17, Lys-23, Leu-38, and Cys-39 were completely conserved in the type-II MADS-box domains, and another 18 residues were also highly conserved (>90%), including Met-1, Arg-3, Lys-9, Ile-11, Gln-18, Val-19, Thr-20, Phe-21, Arg-24, Arg-25, Gly-27, Leu-28, Lys-30, Lys-31, Ala-32, Glu-34, Asp-40, Phe-48, and Ser-49. In addition, we found that the Leu-38 was completely conserved in M_β_ MADS-box domains. Cys-39 was also highly conserved in type-I MADS-box domains, except in M_α_. As with its counterparts in *Arabidopsis*, the lightly conserved Cys-39 residue in M_α_ was partially replaced by Ser. Similarly, Lys-24, Arg-25, and Lys-32 residues were relative highly conserved (>90%) in both type-I and type-II proteins.

### Phylogenetic analysis of BnMADS proteins

To determine the evolutionary relationship of *BnMADSs* between *B*. *napus* and *Arabidopsis*, a neighbor-joining (NJ) phylogenetic tree was constructed based on the alignment of 307 and 107 MADS-box domains in *B*. *napus* and *Arabidopsis*, respectively, using bootstrap values (1000 iterations).

**Fig 2:**
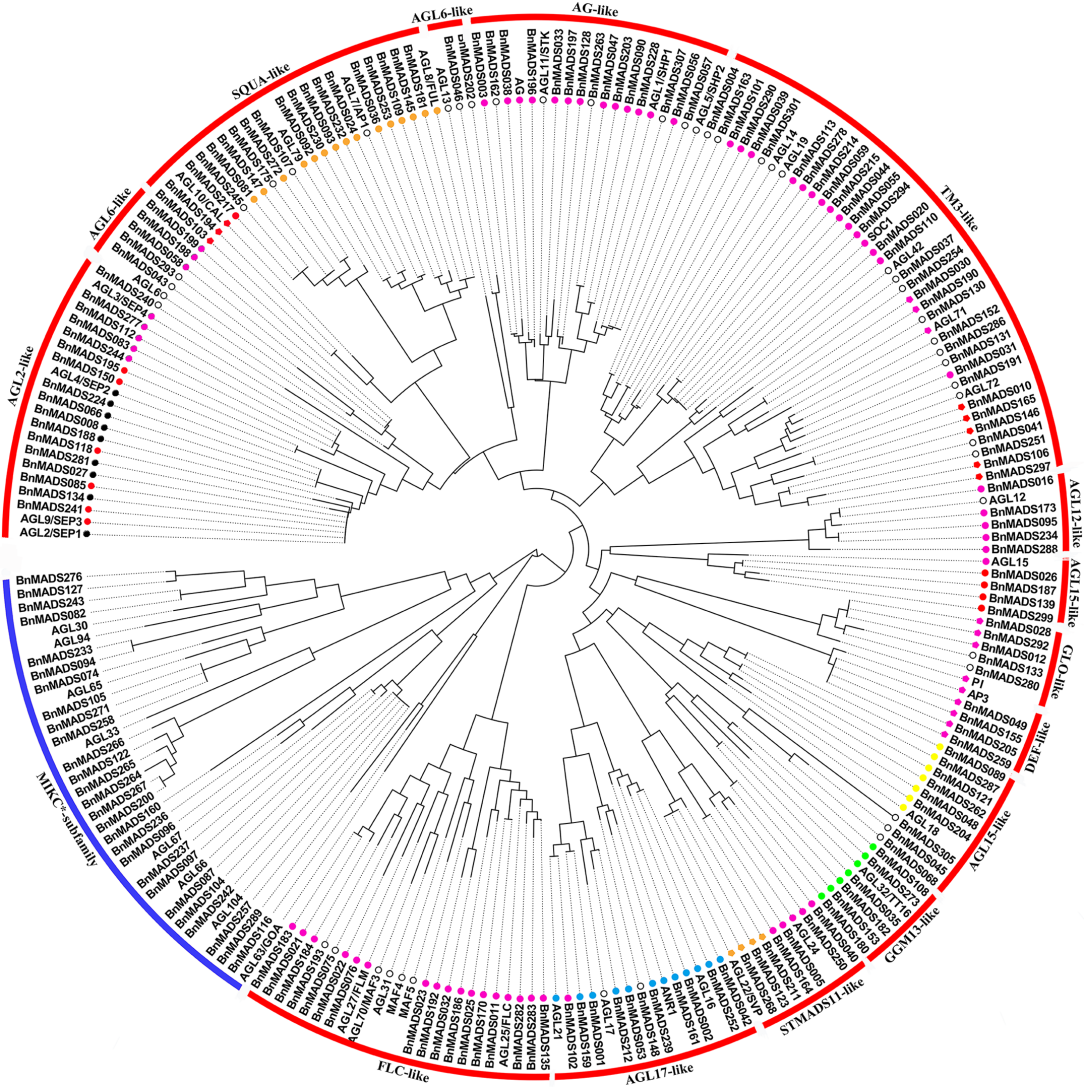
Phylogenetic relationships of type II BnMADS proteins investigated in this study. A neighbor-joining tree representing relationships among 187 BnMADS proteins translated from *B*. *napus* and 45 from *Arabidopsis* are shown. The proteins are clustered into 16 subfamilies. Coloured dots indicate the corresponding intron distribution patterns, as shown in Fig 3.

The 414 MADS-box proteins were divided into two obvious lineages; specifically, type-I and II, with 127 and 180 genes, respectively. Six *BnMADSs* homologous for *AGL33* were assigned to the MIKC* subgroup based on the findings of research on the *Arabidopsis* MADS-box gene family [28]. To better examine the phylogenetic relationships between these two individual lineages, we re-constructed the phylogenetic trees of type I and type II proteins (Fig 2 and S1 Fig). Type-I genes made up a smaller percentage (37.5%) in *B*. *napus* than in *Arabidopsis* (64%), with significantly more type-II genes than type-I genes. This result is consistent with the trend observed in other species [28, 29] and may result from differences in gene-loss bias between these two species.

Based on our results, we grouped the 120 type-I *BnMADSs* into M_α_, M_β_, and M_γ_ clades containing 57, 30, and 33 genes, respectively. The remaining 187 MIKC genes (type II) were classed into MIKC* and MIKC^c^ subfamilies, containing 25 and 161 genes, respectively. Among the 161 MIKC^c^ genes, BnMADS116 and BnMADS289, along with the homologous *AGL63*, could not be assigned to any MIKC^c^ subfamily, possibly attributable to high sequence divergence in the conserved regions, which is consistent with the situation observed in *Arabidopsis* [28]. The MIKC^c^ subfamily was further divided into 13 clades with at least 57% bootstrap support, including AGL2-like, AGL6-like, SQUA-like, AG-like, TM3-like, AGL12-like, AGL15-like, GLO-like, DEF-like, GGM13-like, STMADS11-like, AGL17-like, and FLC-like with 17, 7 19, 15, 33, 5, 13, 5, 4, 6, 7, 11, and 17 members, respectively. Our results indicate that *B*. *napus* and *Arabidopsis* MADS-box proteins were not equal within a given clade, and we commonly observed two or more putative orthologous *BnMADSs* of a single *Arabidopsis* gene. Genes from the AGL6-like and AGL15-like clades were separated into two clades in our phylogenetic tree, indicating that a possible functional divergence appeared between them.

### Intron patterns of *BnMADSs*

Intron and exon structures are important clues to understand the evolutionary gene relationship and functional diversification within a gene family [40]. In the present study, the intron and exon patterns were determined by comparing the full length CDS and DNA sequences of candidate *BnMADSs* using the GSDS web server.

We observed a very striking bimodal distribution of introns between types I and II in the *BnMADSs* family (S1 Table). Type-I genes had fewer intron insertions, mostly varying from 0 to 2, except for *BnMADS077* and *BnMADS156* which had three and *BnMADS300* which had six introns. In contrast, type-II genes had more introns, ranging from 0 to 15, with an average of 5.95. The *BnMADS005* gene had 15 introns, which is the maximum number. Type-II genes with few introns usually did not have *Arabidopsis* orthologs. Candidate MADS-box genes identified from other species such as *Arabidopsis* and *B*. *rapa* also showed the same bimodal distribution of introns [28, 29, 41–43], indicating that this trend is evolutionarily conserved. Moreover, the entire MADS-box domain was located in the first exon, accompanied by an intron insertion with phase 2 in type II genes (S2 Fig).

**Fig 3:**
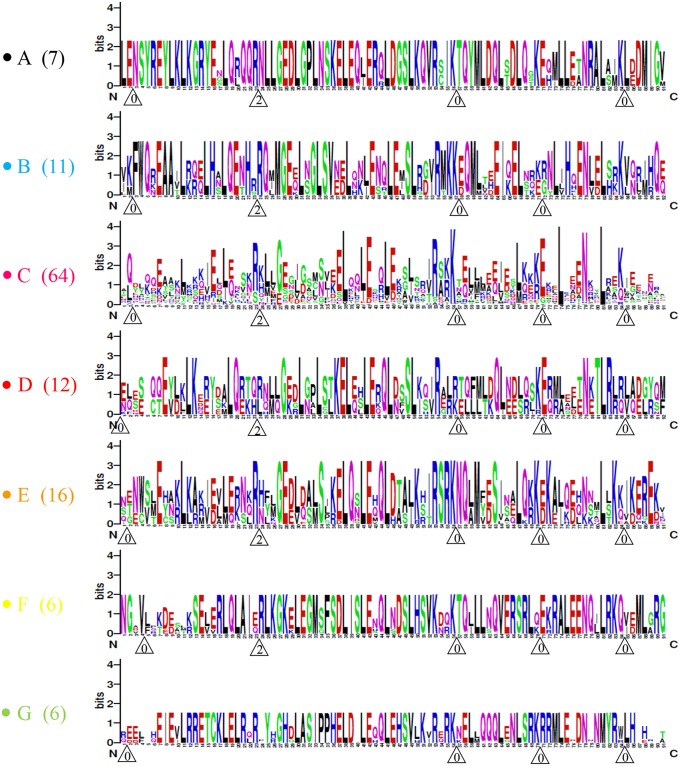
Schematic diagram of intron distribution patterns within the K-box of proteins translated from type II *BnMADSs*. Alignment of the K-box domains is representative of 7 intron patterns, designated A to G. Intron locations are indicated by white triangles, and the number within each triangle indicates the splicing phases: 0 refers to phase 0; 1 to phase 1; and 2 to phase 2. The number of *BnMADSs* within each pattern is presented on the left. The correlation between intron distribution patterns and phylogenetic subfamilies is provided in Fig 2.

The K-box was the second conserved domain of the MIKC^c^ genes, with 4–5 intron insertion sites generally conserved. A total of 7 highly conserved intron patterns (A–G) were first identified in terms of conserved intron insertion sites and phases in this study. Among these conserved intron patterns, C was the main type with the largest number, accounting for 52.5% of type II genes; pattern A accounted for 5.7%, B for 9.0%, D for 9.8%, E for 13.1%, F for 4.9%, and G for 4.9% (Fig 3). Intron insertion sites were conserved in each type, varying from 4 to 5 in different types. Except for the first intron insertion site, the other 4 insertion sites were conserved in all types, with the type A and G lacking the second and fifth intron, respectively. Moreover, amino acids near the intron insertion site were somewhat conserved (Fig 3). Interestingly, in the phylogenetic tree, genes in the same clade generally shared the same intron patterns (Fig 2). This finding constitutes an independent criterion for testing the reliability of our phylogenetic analysis.

### Common motifs identified in the non-conserved domain supports subgroup designation

To discover motifs shared among related proteins within subfamilies, the MEME tool was used to analyse the relatively non-conserved regions of *BnMADSs*. Since regions following MADS-box domains in type-II BnMADS proteins are well known as the I-domain, K-box, and C-terminal, we only included type-I BnMADS proteins in the MEME analysis.

**Fig 4:**
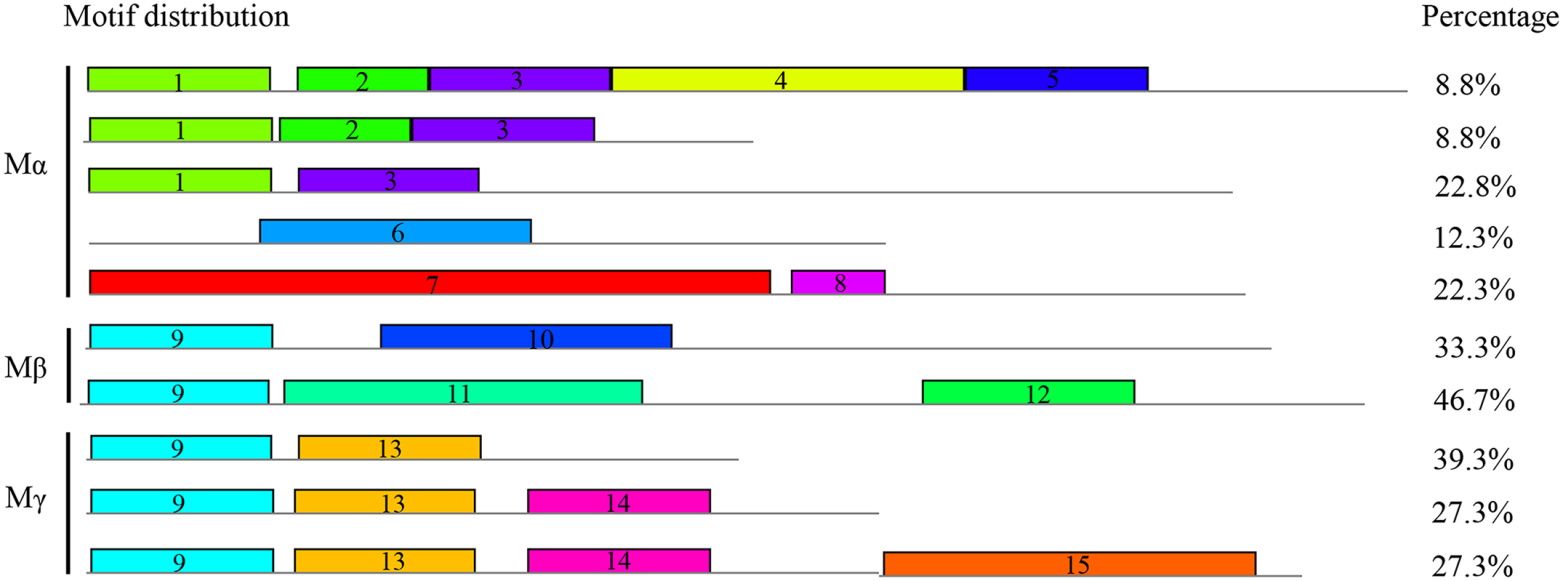
Architecture of conserved protein motifs translated from type I *BnMADSs*. The percentage of BnMADS proteins with each pattern is presented on the right. Numbers in the colored bar indicate different motifs.

In total, 15 conserved motifs of variable length (15–108 amino acids) were detected in the C-terminals of type I proteins (S2 Table). We found that members of the same clade generally shared one or more motifs (Fig 4). For example, 8 motifs (1–8) were identified in M_α_, which encompassed 40.4%, 17.6%, 40.4%, 8.8%, 8.8%, 12.3%, 22.3%, and 22.3% respectively; M_β_ protein members contained motifs 9–12, with up to 90% contained motif 9; members of M_γ_ contained motif 9 and 14–15. In general, most of these 15 motifs were clade-specific rather than subfamily-specific, except motif 9, which was shared by M_β_ and M_γ_, supporting the evolutionary relationship of each subfamily. However, the function of these motifs remains unknown.

### Chromosomal distribution and duplication events of *BnMADSs*

To investigate the relationship between genetic divergence and gene duplication within the *B*. *napus* MADS-box gene family, we analysed the chromosomal locations of *BnMADSs* based on information from the Genoscope genomic database.

**Fig 5:**
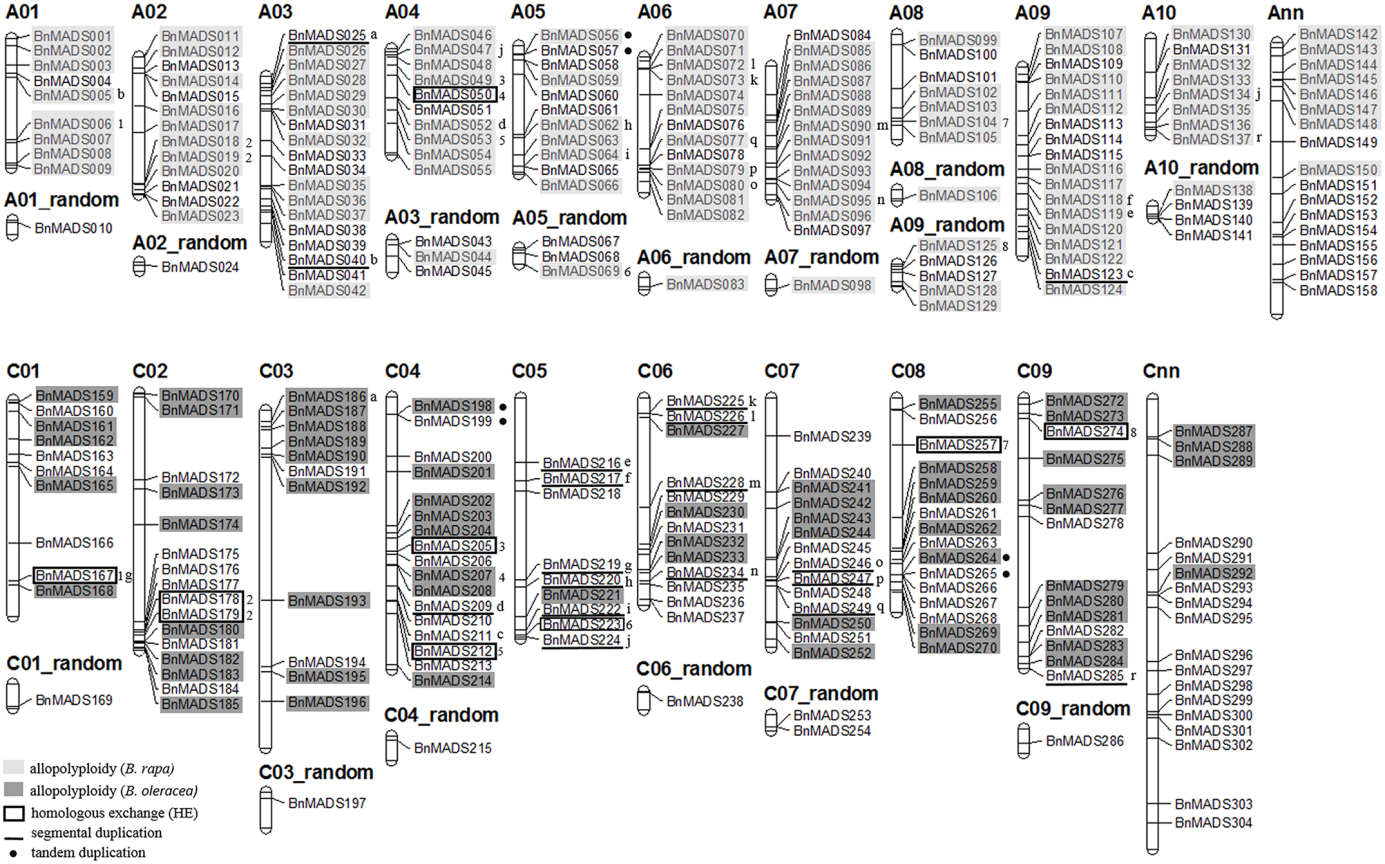
Chromosomal locations and duplications of *BnMADSs*. The chromosome number is indicated above each chromosome. The scale is in megabases (Mb). Genes inherited from *B*. *rapa* or *B*. *oleracea* genomes are under grey and black backgrounds respectively; genes originating from segmental duplication are underlined by black lines accompanied with the same letter (e.g., a, b, and c); genes arising from homologous exchange (HE) are indicated using black boxes accompanied with the same number (e.g., 1, 2, and 3); and genes originating from tandem duplication are marked with black dots.

Results show that *BnMADSs* were distributed across all 19 chromosomes, and the number of genes within each sub-genome appeared to be uneven (Fig 5). The A_n_-subgenome had an average of 12.1 *BnMADS* on its 10 chromosomes; A08 had a minimum number of 7, while A03 and A09 had a maximum of 18 genes. The average number of *BnMADSs* in the C_n_-subgenome was 13.3, with the C05 containing a minimum of 9 genes and the C04 having as many as 17 genes. Thus, there is not a biased tendency between these two subgenomes. Moreover, the distribution of candidates on each chromosome was not quite balanced, except for A07 and A09; the genes tended to distribute on the terminal parts. This pattern was similar to the MADS-box genes observed in *B*. *rapa* and *Arabidopsis* [28].

A total of 201 *BnMADSs* were identified to have syntenic relationships, of which 174 *BnMADSs* were inherited from *B*. *rapa* or *B*. *oleracea* genomes (S3 Table). In contrast, only 18 genes from 18 pairs originated from segmental duplication in the *B*. *napus* genome, and 3 genes from 3 pairs were from tandem duplication. Among those genes from segmental duplications, 11 of 18 genes belonged to the type-I subfamily, while the remaining 7 genes belonged to type-II, confirming a higher frequency of segmental gene duplication in the type-I subfamily [44]. Moreover, according to the distribution on chromosome and sequence similarity, *BnMADS056/057*, *BnMADS198/199*, and *BnMADS264/265* were identified as tandem duplication gene pairs.

Together, genes obtained from tandem duplication (3) and segmental duplication (18) took up 0.98% and 5.86% of the BnMADS gene family, respectively, providing an outstanding example of genome-wide allopolyploidization between *B*. *rapa* and *B*. *oleracea* that mainly contributed to the large *BnMADSs* expansion in *B*. *napus* (56.68%).

### Cis-acting regulatory elements in the *BnMADSs* promoters

Cis-elements in the promoter of a gene can regulate the initiation and efficiency of gene transcription by binding with transcription factors. The cis-acting regulatory elements of *BnMADS* promoters were predicted using PlantCARE.

**Fig 6:**
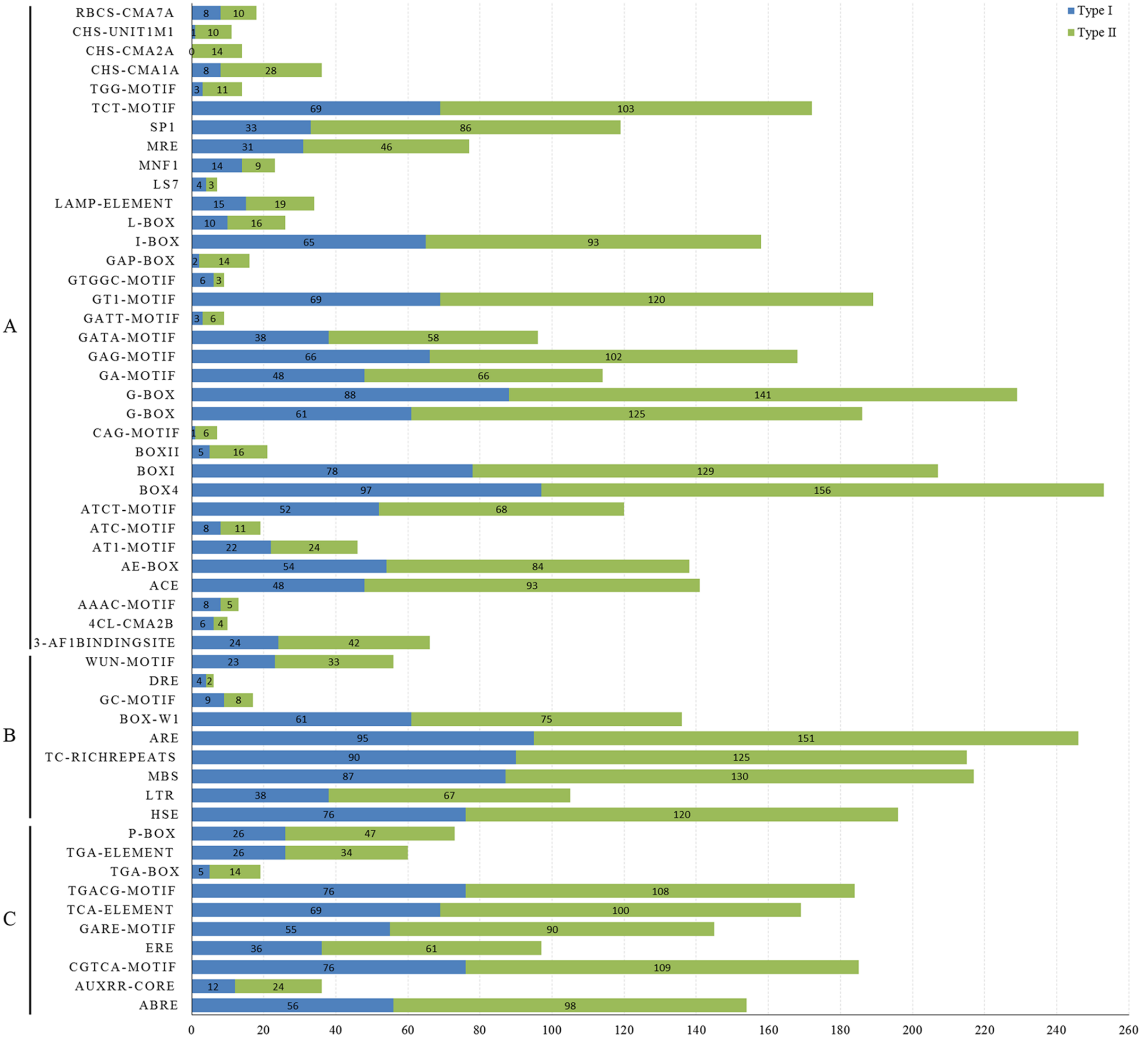
Classification of the cis-elements in *BnMADS* promoters. 106 Cis-elements were identified in the promoters of all 307 *BnMADSs* and were classified into three main groups (A–C). The number of type I and type II genes with the same type of cis-element is marked in different colours.

In all, three cis-element groups in *BnMADS* promoters were classified (A–C) depending on their functional annotations (Fig 6 and S4 Table). The A group contained elements responding to light, the B group consisted of elements responding to biotic and/or abiotic stress induction, and the C group contained elements responding to hormones.

In the A group, 34 elements were related to the light response, implying that *BnMADSs* expression is induced by light, which is consistent with the function of many MADS-box genes in plants that use a fluorescent process widely regulated by light. Elements in the B group consisted of many stress response elements, such as MYB transcription factor binding site (MBS, drought stress response element), heat shock element (HSE, heat stress response), and TC-rich repeats (stress reaction), indicating that *BnMADSs* may respond to abiotic stress responses. There are some cis-elements in the C group, including the salicylic acid (TCA) response element, gibberellin response element (GARE-motif), CGTCA-motif/TGACG-motif (methyl jasmonate [MeJA] response), and abscisic acid response (ABRE), accounting for over 50% of both type-I and type-II genes and indicating possible roles for candidate genes in hormone response processes.

### Genome-wide expression of *BnMADSs* in *B*. *napus* by RNA-seq

Gene expression patterns have been examined in relation to gene function [45], therefore, we investigated the expression of all 307 *BnMADSs* in 50 different tissues at different stages.

**Fig 7:**
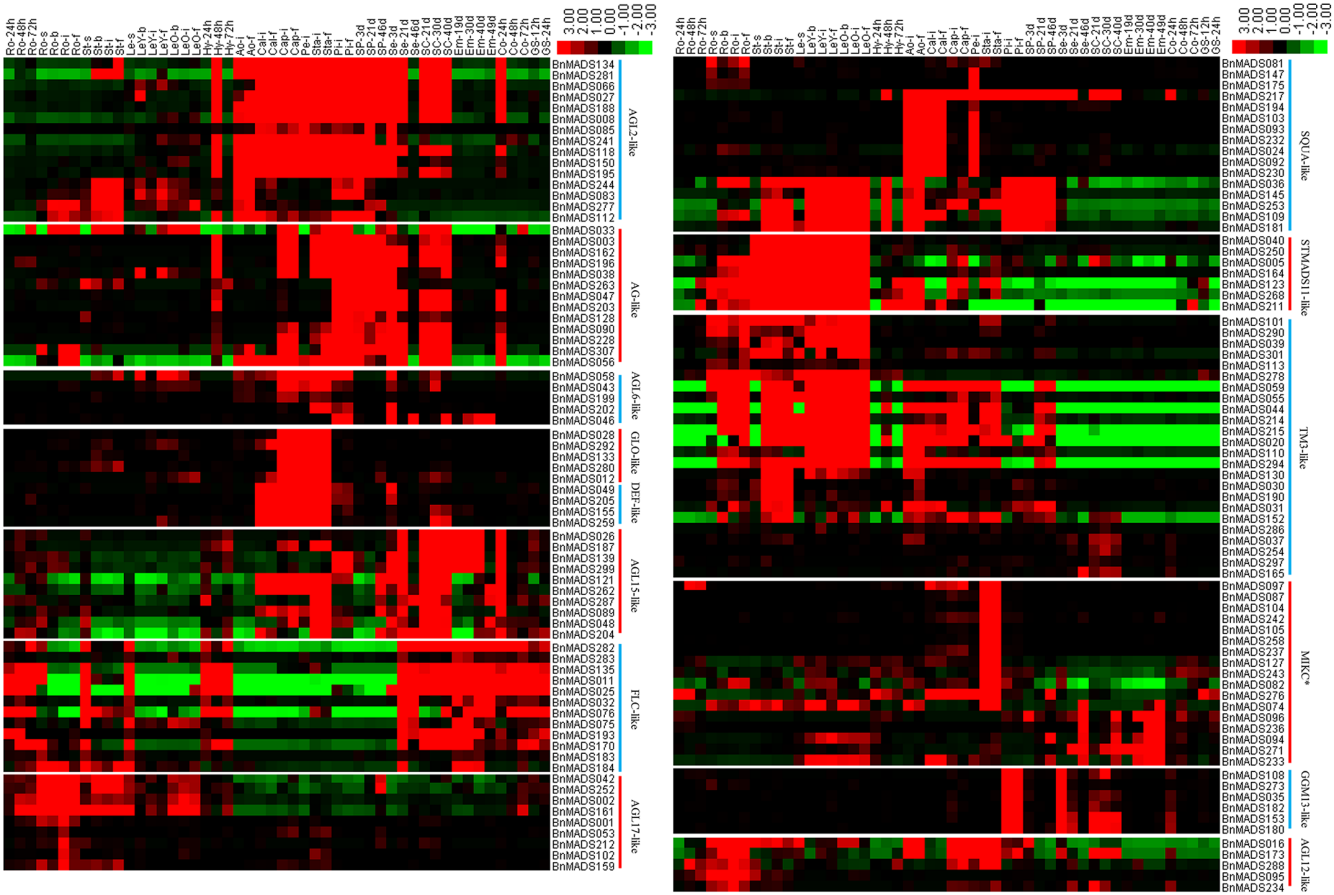
Expression profiles of type II *BnMADSs* across different developmental stages and organs. The genes and their corresponding clade are on the right. The tissues used for expression analysis are indicated at the top of each column. GS, germinate seed; Hy, hypocotyl; Ao, anthocaulus; Ro, root; St, stem; Le, leaf; Cal, calyx; Cap, capillament; Pe, petal; Sta, stamen; Pi, pistil; SP, silique; Se, seed; SC, seed coat; Em, embryo; Co, cotyledon. s, seedling stage; b, bud stage; i, initial flowering stage; and f, full-bloom stage. The time after seed germination is indicated as 24, 48, and 72 h. The number of days after pollination (DAP) is indicated as 3, 19, 21, 30, 40, and 46 d. The colour bar represents log2 expression values (FPKM). The genes with weak or no expression are supplied in S1 Table.

As shown in Fig 7, most *BnMADSs* were differentially expressed in different tissues in both vegetative and reproductive organs at different stages, where the type II genes had much higher and broader expression patterns. In contrast, the majority of type I genes had no or very low expression levels, with a few exceptions, indicating that type II genes may have abundant functions while type I genes have limited functions or are only expressed under special conditions in *B*. *napus*, such as stress and induction of hormones.

In the type II subfamily, 20 of 187 (10.69%) genes were not expressed in any tissues, 18 (9.63%) genes had very weak expression levels (S1 Table), and the expression patterns of the remaining 149 (79.68%) were clustered into three main blocks, with some genes (38, 25.5%) having higher expression levels in the root, stem, and leaf during the seedling, budding, and flowering stages (Fig 7). Furthermore, some genes (83, 55.7%) had relatively higher expression levels in the flower and/or early developmental seed tissues, while some (28, 18.79%) had higher expression levels in the seed tissues 19 days after pollination (DAP). It is obvious that the main function of *BnMADSs* may involve reproductive organs (flower and/or seed tissues). Moreover, the expression patterns of *BnMADSs* in different tissues of the same organ, such as the flower and seeds, were generally different. For example, highly expressed genes belonging to the GGM13-like clade were primarily in female-reproductive related organs such as the pistil, and highly expressed members of the MIKC* subfamily primarily occurred in male-reproductive organs such as the stamen. However, homologous genes in the same subfamily usually had a conserved expression profile, suggesting functional redundancy. For instance, most members of the AGL2-like clade were highly expressed in flowers and seed tissues during the early development stage, and members of the STMADS11-like clade were highly expressed in the root and/or stem and leaves.

In the type-I subfamily, 17 out of 120 (14.17%) genes were not expressed in any tissues, 84 (70%) had very low expression levels (S1 Table), and 19 (15.83%) showed relatively high expression levels in *B*. *napus* (S3 Fig). Moreover, expression patterns of the genes with high expression levels were broad in terms of reproductive and vegetable organs and were not conserved between genes. In addition, the entire M_β_ subfamily of genes showed very low expression levels.

Taken together, the type II genes had much wider and higher expression levels than type I genes in *B*. *napus*, and the expression levels were generally conserved in each subfamily but were different across distinct subfamilies.

## Discussion

### A good example of gene expansion and loss of the MADS-box gene family in land plants

In the present study, we identified 307 MADS-box members in the *B*. *napus* genome (*BnMADSs*), which is the largest number reported in plants to date [28, 29, 41–43]. Among the identified genes, type-I genes made up 37.5% of the *BnMADSs* gene family, while in *Arabidopsis* the percentage is 64% [28]. In the case of *B*. *rapa* and *B*. *oleracea*, 40% [29] and 34% of MADS-box genes are type I, respectively. Similarly, in higher plants such as rice, maize, soybean, and sorghum, the percentage of type II genes is much higher than type I genes [41, 42, 46]. In lower land plants such as *Selaginella*, this trend is similar to that observed in *Arabidopsis* [47]. Overall, it is obvious that the percentage of type-I genes generally diminishes during the evolution to higher plants. The functions of type-I genes have been minimally reported to date; however, we speculated that the presence of many non-functional type-I genes (i.e., pseudogenes) indicates inactivity. In contrast, the number of type II genes in higher plants obviously increased, and type-II genes appear to have differentiated functionally in a relatively short time, likely attributable to type II genes being maintained as functional genes in the genome to perform more complex functions during flower and organ development [44].

Type-I and II genes differentiated in duplication patterns. According to synteny analysis, 201 out of 307 *BnMADSs* had syntenic relationships with MADS-box genes from *B*. *rapa* or *B*. *oleracea*, respectively, proving that the majority of *BnMADSs* were obtained from inheritance and genome-wide allopolyploidy of *B*. *rapa* and *B*. *oleracea* genomes. Among these, 57 type-I genes (47.5%) come from allopolyploidy and 11 genes (9.17%) are segmental duplications in *B*. *napus*. In contrast, 117 type-II genes come from allopolyploidy (62.57%), while 7 genes (3.74%) and 3 genes (1.6%) are segmental and tandem duplications, respectively.

In addition, there were 109 and 90 syntenic genes in the A_n_- and C_n_-subgenomes, respectively. After allopolyploidy, the gene retainment ratio was mainly balanced between these two subgenomes, with the A_n_-subgenome slightly advantaged. Furthermore, homologous exchange (HE), meaning replacement of a chromosomal region with a duplicated copy from the corresponding homologous subgenome, was found to be frequent between *B*. *napus* subgenomes [48]. Accordingly, we identified 18 genes from 8 pairs located in known HE blocks [48], and the number of type I genes (6, 5%) was higher than the number of type II genes (3, 1.6%), indicating HE was an important source underlying the evolution of *BnMADSs* after allopolyploidization, especially for type I genes. Meanwhile, among HE events, more genes from the Cn-genome were replaced by genes from the An-genome, indicating that genes from the latter were more retained.

Overall, genome-wide allopolyploidy, segmental duplication and HE were the main expansion mechanisms of *BnMADSs*, and subsequent duplications were preferred in the type-I genes over the type-II genes in the *B*. *napus* genome.

### An ignored evolutionary story of intron insertion patterns in the K-box domain

Many studies have revealed the conservation of intron insertion sites in relatively conserved DNA binding domains of many transcription factor gene families [49, 50]. Similarly, nearly all studies of the MADS-box gene family focus on the MADS-box domain. In this study, we found that all MADS-box domains of the BnMADS gene family are in the first exon, with no intron insertions observed in this domain.

Interestingly, we found that the intron insertion patterns in the K-box domain are highly conserved in type-II genes, and type-II genes could be further classified into 7 conserved intron patterns in terms of absolute conserved intron insertion positions and phases (A–G types) (Fig 3). The gene structure and sequence characteristics of these 7 types were highly similar, indicating a relatively close relationship. There were 4–5 conserved introns inserted into the K domain in each type. While the location of the first intron was variable across different gene types, the remaining were highly conserved. Genes in the MIKC^c^ clade were excluded in the analysis, attributable to an incomplete K-box domain. Interestingly, as discussed below, we found that genes in the MIKC^c^ clade were not expressed.

To verify the intron patterns in different species of land plants, we extended this analysis to 19 other species, including chlorophytes (*Chlamydomonas reinhardtii* and *Volvox carteri*), embryophytes (*Marchantia polymorpha*, *Physcomitrella patens*, and *Sphagnum fallax*), tracheophytes (*Selaginella moellendorffii*), basic angiosperm (*Amborella trichopoda* and *Zostera marina*), monocots (*Brachypodium distachyon*, *Oryza sativa*, *Sorghum bicolor*, and *Zea mays*), and eudicots (*Sorghum bicolor*, *Vitis vinifera*, *Populus trichocarpa*, *Citrus sinensis*, *Glycine max*, and *Malus domestica*) (S5 Table). We found that the C type was distributed in all species investigated except chlorophytes, while the A and B types existed in some embryophyte species, indicating that the C type is older. The E type was distributed in basic angiosperms (*Amborella trichopoda* and *Zostera marina*), indicating it is relatively older than the last three types (D, F, and G). The D and G types were found in all flowering plants, including monocots and dicots, suggesting they may predate divergence of these two lineages. F types were only distributed in the Brassica species investigated in this study, indicating a new origin in this lineage. These results proved that the intron patterns in the K-box are highly conserved across land plants, thus should be a typical feature for the MADS-box gene family.

In the phylogenetic tree, the intron patterns of *BnMADSs* were generally conserved within most of the clades, except for genes lacking a full ORF, suggesting a common origin of members in each clade. For example, members of the AGL17-like clade shared the B intron pattern, while members of the AGL15-like clade shared the F type (Fig 2). However, this pattern was not absolute, as the intron patterns of the AGL2-like clade were mixed, containing both the A and D types. Given that type A exists in lower land plants and members having similar functions to those of pattern D in flower development, we speculated that type D likely developed from type A. Interestingly, it has been reported that *AGL18* (type F) and *AGL15* (type C) are functionally redundant in floral repress [51] and could be classified into one clade [28]. However, in our phylogenetic tree, the homologous nature of these two groups included different intron patterns; therefore, we divided them into two clades (AGL15-like and AGL18-like clades). This result proves that intron patterns support classification of the phylogenetic tree. Type F existed only in Cruciferae plants, while pattern C was widely distributed in all species investigated; these results indicated that type F originated from type C in the *Brassica* lineage.

Furthermore, the function of the A, C, D, and E types relates to the flower and fruit [51–59]; the B type relates to root development [60] and seeds [61]; and the G type is important for the colour of the seed epidermis [62], which likely explains the closer relationship between the A, C, D, and E types while the B and G types were more independent. It is also interesting to note that there are 65 C-type *BnMADSs*, which is higher than the number of other types in *B*. *napus* (Fig 3). Similar results were also observed in all the flowering plants investigated in this study (S5 Table). Further, the B pattern, which is related to root development, took up the highest percentage in *Selaginella*. These phenomena support our hypothesis that the intron insertion pattern is related to a certain function in land plants.

### Functional conservation and redundancy of *BnMADSs* based on expression analysis

Our results show that the expression of *BnMADSs* is mainly conserved in each family and is very similar to that of the homologous genes in *Arabidopsis*. These results suggest functional conservation of homologous genes across different plants. For example, the genetic and molecular basis of floral development have revealed that numerous MADS-box genes are involved in specifying floral organ identity in *Arabidopsis* [11, 58, 62–64]. Accordingly, the *Arabidopsis SEP1/2/3* genes in the AGL2-like clade were identified as class E genes and function as organ-identity genes in development of the petal, stamen, and carpel. Similarly, *BnMADSs* in the AGL2-like clade are primarily highly expressed in the flower (calyx, capillament, petal, stamen, and pistil) and seed tissues during the early development stages (seed and seed coat) (Fig 7). The short vegetative phase (*SVP*) gene in the STMADS11-like clade was identified as a floral repressor and plays an essential role in determining the length of vegetative growth; *BnMADSs* in the same clade are all highly expressed in vegetative organs at different stages. The transparent Testa16 (*TT16*) gene in the GGM13-like clade is involved in the determination of seed coat colour; *BnMADSs* in this clade are primarily highly expressed in the female-reproductive organs (pistil) and seed tissues during the early stages. Trends in the other subfamilies were the same.

Moreover, 65 pairs of 142 type II paralogous genes were identified in this study (S6 Table), including 56 pairs with two genes (six genes had some deletions in the ORF regions), six pairs with three genes, and three pairs with four genes. While six genes had some deletions in the ORF region, the last of the 50 pairs contained two genes and shared a very high degree of sequence identity or similarity in the binding domain and full protein length, with an average identity of approximately 99% in the binding domain and an average identity of 85% in the full-length proteins. In addition, the DNA binding domains of 42 out of the 65 pairs were the same (Fig 7). Accordingly, most of the paralogous *BnMADSs* shared a similar or the same expression patterns, implying a functional redundancy. For example, *BnMADS103/194*, *BnMADS030/190*, *BnMADS045/068*, *BnMADS035/182*, and *BnMADS153/180* showed the same expression pattern. Furthermore, the expressions of sister pair genes showed no bias, as candidates from the A_n_- and/or C_n_-subgenome were quite similar. For instance, *BnMADS092/230* contained two genes from the A_n_- and C_n_-subgenome and shared a similar expression pattern, while *BnMADS045/068* contained two genes from the A_n_-subgenome and had nearly the same expression patterns. In addition, some sister pairs may represent neo-functionalization or sub-functionalization, as they showed similar expression patterns with slight diversification. For instance, *BnMADS150/195* were both highly expressed in the calyx, capillament, and petal; however, *BnMADS150* was highly expressed in the pistil, whereas *BnMADS195* was not (Fig 7). The expression of several pairs was obviously different, which may indicate functional divergence; *BnMADS032* from the *BnMADS032/192* gene pair was highly expressed in vegetative organs and seeds, while *BnMADS192* was not expressed. As mentioned above, the 20 type II *BnMADSs* that were not expressed in any tissues investigated were generally the homologous genes in a given clade; five were duplications and/or sister pair genes (S3 and S6 Tables). These data suggested functional redundancy of highly homologous genes.

Furthermore, sequence analysis showed that the promoter regions of sister pairs were also highly homologous, with the average sequence identity ranging from 48% to 97% (S6 Table). In general, expression patterns and promoter sequence identity are closely related. Expression of 55 of the 65 pairs was similar or the same, indicating functional redundancy; the last generally had more sequence identity in the ORF regions, but less in the promoter regions, suggesting that functional divergence may first occur in the promoter region of sister pairs during evolution.

Thus, our data show that expression patterns of homologous genes in a given subfamily or sister pair are generally conserved, revealing a major functional conservation as well as redundancy of *BnMADSs* during evolution.

## Conclusions

Results of the present work confirm many features described in other species. However, some novel characteristics were found in the *B*. *napus* MADS-box gene family, including intron patterns in the K-box and expansion mechanisms after allopolyploidy between *B*. *rapa* and *B*. *oleracea*. Further, our phylogenetic analyses provide a useful reference for postulating functional hypotheses for uncharacterized *BnMADSs*. In this study, we evaluated the roles and evolution of uncharacterized MADS-box genes in *B*. *napus* using a comparative phylogenetic analysis of *B*. *napus*, *B*. *rapa*, *B*. *oleracea*, and *Arabidopsis*, as well as information from previous research.

## Supporting information

S1 Fig Phylogenetic relationships of type I MADS-box proteins (*BnMADSs*) investigated in this study. A neighbor-joining tree representing relationships among 120 type I MADS-box proteins from *B*. *napus* and 63 from *Arabidopsis* is shown. The proteins are clustered into 3 clades, including M_α_, M_β_, and M_γ_.
(PDF)

S2 Fig Structures of the *BnMADSs* in *B*. *napus*. Exon(s) are indicated by yellow boxes, MADS-box domain(s) by orange boxes, untranslated region(s) by grey boxes, and spaces between the coloured boxes correspond to introns. Exon and intron size can be estimated using the horizontal scale bar. (A): the structures of type I *BnMADSs*; (B): the structures of type II *BnMADSs*.
(PDF)

S3 Fig Expression profiles of *B*. *napus* MADS-box genes (*BnMADSs*) across 50 different developmental stages and organs. The tissues used for expression analysis are indicated at the top of each column. GS, germinate seed; Hy, hypocotyl; Ao, anthocaulus; Ro, root; St, stem; Le, leaf; Cal, calyx; Cap, capillament; Pe, petal; Sta, stamen; Pi, pistil; SP, silique; Se, seed; SC, seed coat; Em, embryo; Co, cotyledon. s, seedling stage; b, bud stage; i, initial flowering stage; and f, full-bloom stage. The time after seed germination is indicated as 24, 48, and 72 h. Days after pollination (DAP) are indicated as 3, 19, 21, 30, 40, and 46 d. The colour bar represents log2 expression values (FPKM). Genes with weak or no expression are supplied in S1 Table.
(PDF)

S1 Table MADS-box genes identified in *B*. *napus* in this study. (XLSX)

S2 Table Motifs identified in type I BnMADS-box proteins in *B*. *napus* via MEME analysis. (XLSX)

S3 Table Duplications of MADS-box genes in *B*. *napus*, *B*. *rapa*, and *B*. *oleracea*. (XLSX)

S4 Table Cis-element analyses in promoter regions of 307 *BnMADSs*. (XLSX)

S5 Table Intron pattern analyses in the K-box domain of MADS-box genes in 21 plant species investigated in this study. (XLSX)

S6 Table Homology and expression analysis of 59 sister pairs in *BnMADSs*. (XLSX)
