# Supplementary material for: Evolution and expression analyses of the MADS-box gene family in Brassica napus
Source: PLoS One. 2018 Jul 19;13(7):e0200762. doi: 10.1371/journal.pone.0200762 (PMC6053192; doi:10.1371/journal.pone.0200762)
Supplement: S1 Fig — A neighbor-joining tree representing relationships among 120 type I MADS-box proteins from B. napus and 63 from Arabidopsis is shown. The proteins are clustered into 3 clades, including Mα, Mβ, and Mγ. (PDF) [file pone.0200762.s001.pdf]

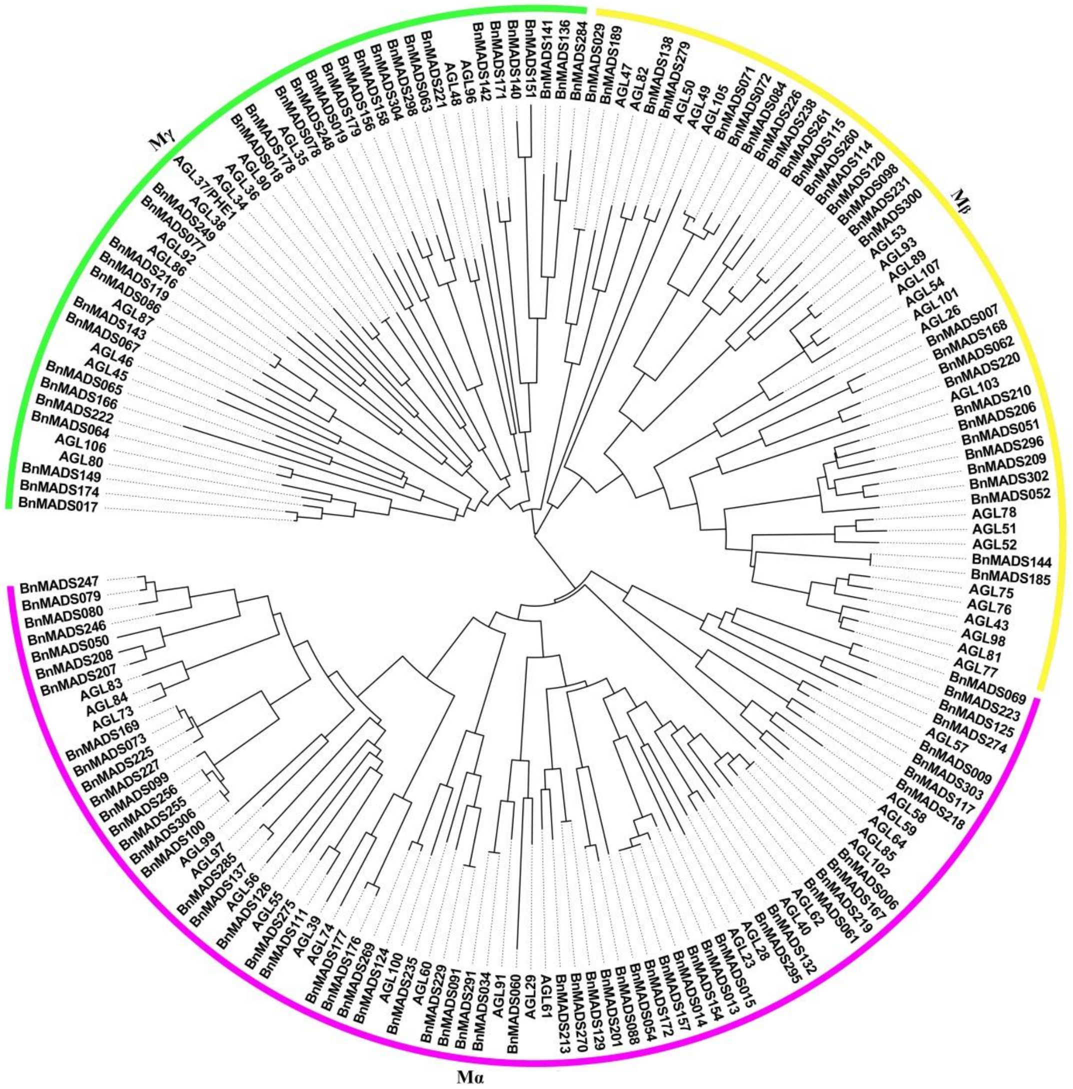

S1 Fig. Phylogenetic relationships of type I MADS-box proteins (*BnMADSs*) investigated in this study. A neighbor-joining tree representing relationships among 120 type I MADS-box proteins from *B. napus* and 63 from *Arabidopsis* is shown. The proteins are clustered into 3 clades, including M_{α}, M_{β}, and M_{γ}.
