# Supplementary material for: Evolution and expression analyses of the MADS-box gene family in Brassica napus
Source: PLoS One. 2018 Jul 19;13(7):e0200762. doi: 10.1371/journal.pone.0200762 (PMC6053192; doi:10.1371/journal.pone.0200762)
Supplement: S3 Fig — The tissues used for expression analysis are indicated at the top of each column. GS, germinate seed; Hy, hypocotyl; Ao, anthocaulus; Ro, root; St, stem; Le, leaf; Cal, calyx; Cap, capillament; Pe, petal; Sta, stamen; Pi, pistil; SP, silique; Se, seed; SC, seed coat; Em, embryo; Co, cotyledon. s, seedling stage; b, bud stage; i, initial flowering stage; and f, full-bloom stage. The time after seed germination is indicated as 24, 48, and 72 h. Days after pollination (DAP) are indicated as 3, 19, 21, 30, 40, and 46 d. The colour bar represents log2 expression values (FPKM). Genes with weak or no expression are supplied in S1 Table. (PDF) [file pone.0200762.s003.pdf]

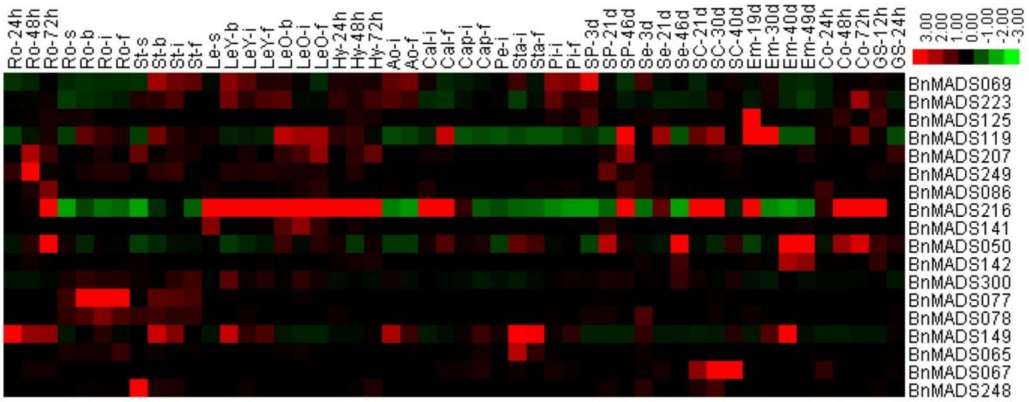

S3 Fig. Expression profiles of *B. napus* MADS-box genes (*BnMADSs*) across 50 different developmental stages and organs. The tissues used for expression analysis are indicated at the top of each column. GS, germinate seed; Hy, hypocotyl; Ao, anthocaulus; Ro, root; St, stem; Le, leaf; Cal, calyx; Cap, capillament; Pe, petal; Sta, stamen; Pi, pistil; SP, silique; Se, seed; SC, seed coat; Em, embryo; Co, cotyledon. s, seedling stage; b, bud stage; i, initial flowering stage; and f, full-bloom stage. The time after seed germination is indicated as 24, 48, and 72 h. Days after pollination (DAP) are indicated as 3, 19, 21, 30, 40, and 46 d. The colour bar represents log2 expression values (FPKM). Genes with weak or no expression are supplied in Table S1.
