# Supplementary material for: Evolution and expression analyses of the MADS-box gene family in Brassica napus
Source: PLoS One. 2018 Jul 19;13(7):e0200762. doi: 10.1371/journal.pone.0200762 (PMC6053192; doi:10.1371/journal.pone.0200762)
Supplement: S2 Fig — Exon(s) are indicated by yellow boxes, MADS-box domain(s) by orange boxes, untranslated region(s) by grey boxes, and spaces between the coloured boxes correspond to introns. Exon and intron size can be estimated using the horizontal scale bar. (A): the structures of type I BnMADSs; (B): the structures of type II BnMADSs. (PDF) [file pone.0200762.s002.pdf]

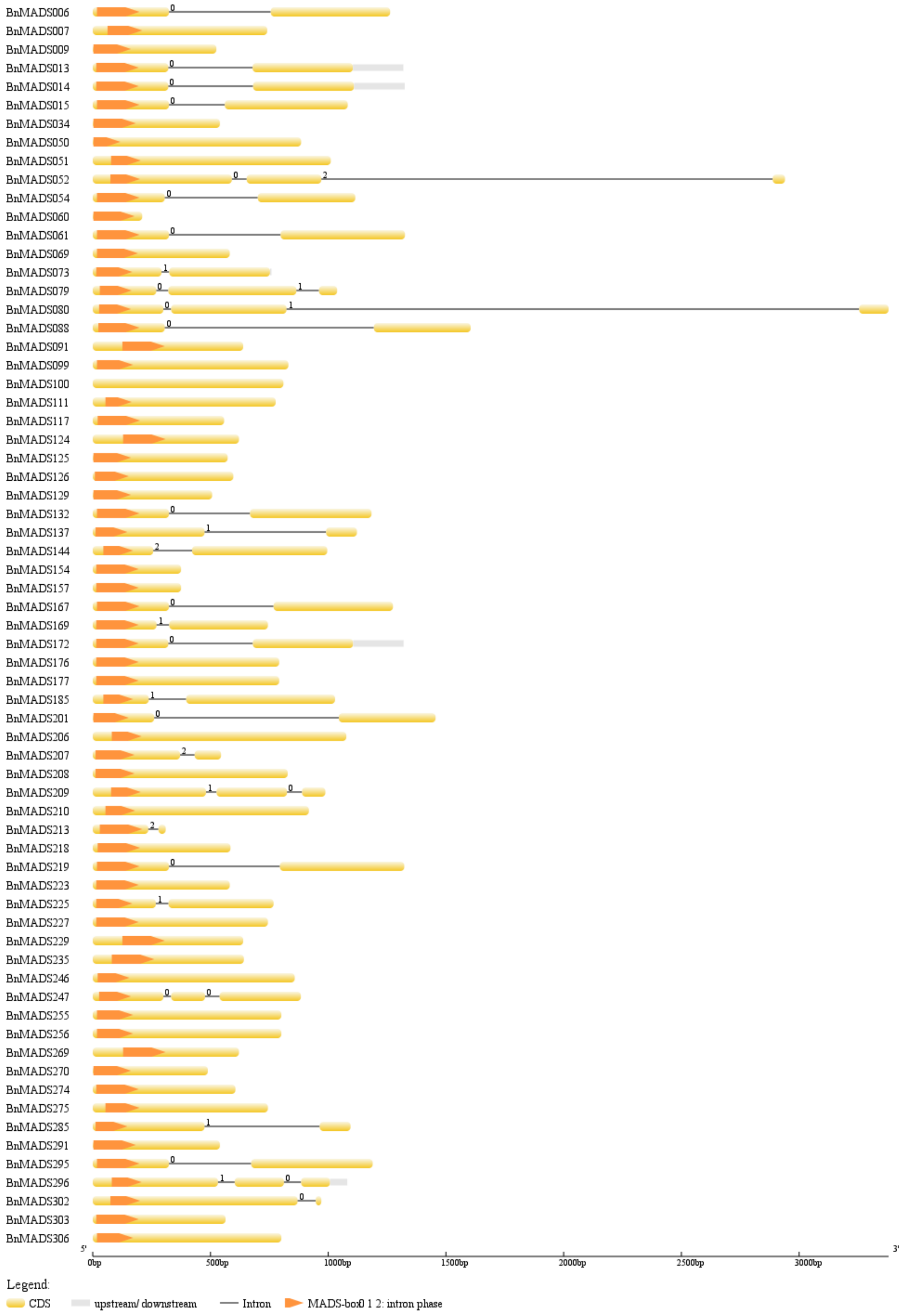
BnMADS006
BnMADS007
BnMADS009
BnMADS013
BnMADS014
BnMADS015
BnMADS034
BnMADS050
BnMADS051
BnMADS052
BnMADS054
BnMADS060
BnMADS061
BnMADS069
BnMADS073
BnMADS079
BnMADS080
BnMADS088
BnMADS091
BnMADS099
BnMADS100
BnMADS111
BnMADS117
BnMADS124
BnMADS125
BnMADS126
BnMADS129
BnMADS132
BnMADS137
BnMADS144
BnMADS154
BnMADS157
BnMADS167
BnMADS169
BnMADS172
BnMADS176
BnMADS177
BnMADS185
BnMADS201
BnMADS206
BnMADS207
BnMADS208
BnMADS209
BnMADS210
BnMADS213
BnMADS218
BnMADS219
BnMADS223
BnMADS225
BnMADS227
BnMADS229
BnMADS235
BnMADS246
BnMADS247
BnMADS255
BnMADS256
BnMADS269
BnMADS270
BnMADS274
BnMADS275
BnMADS285
BnMADS291
BnMADS295
BnMADS296
BnMADS302
BnMADS303
BnMADS306
5'
3'
0bp
500bp
1000bp
1500bp
2000bp
2500bp
3000bp
Legend:
CDS
upstream/ downstream
Intron
MADS-box
0 1 2: intron phase

(A)

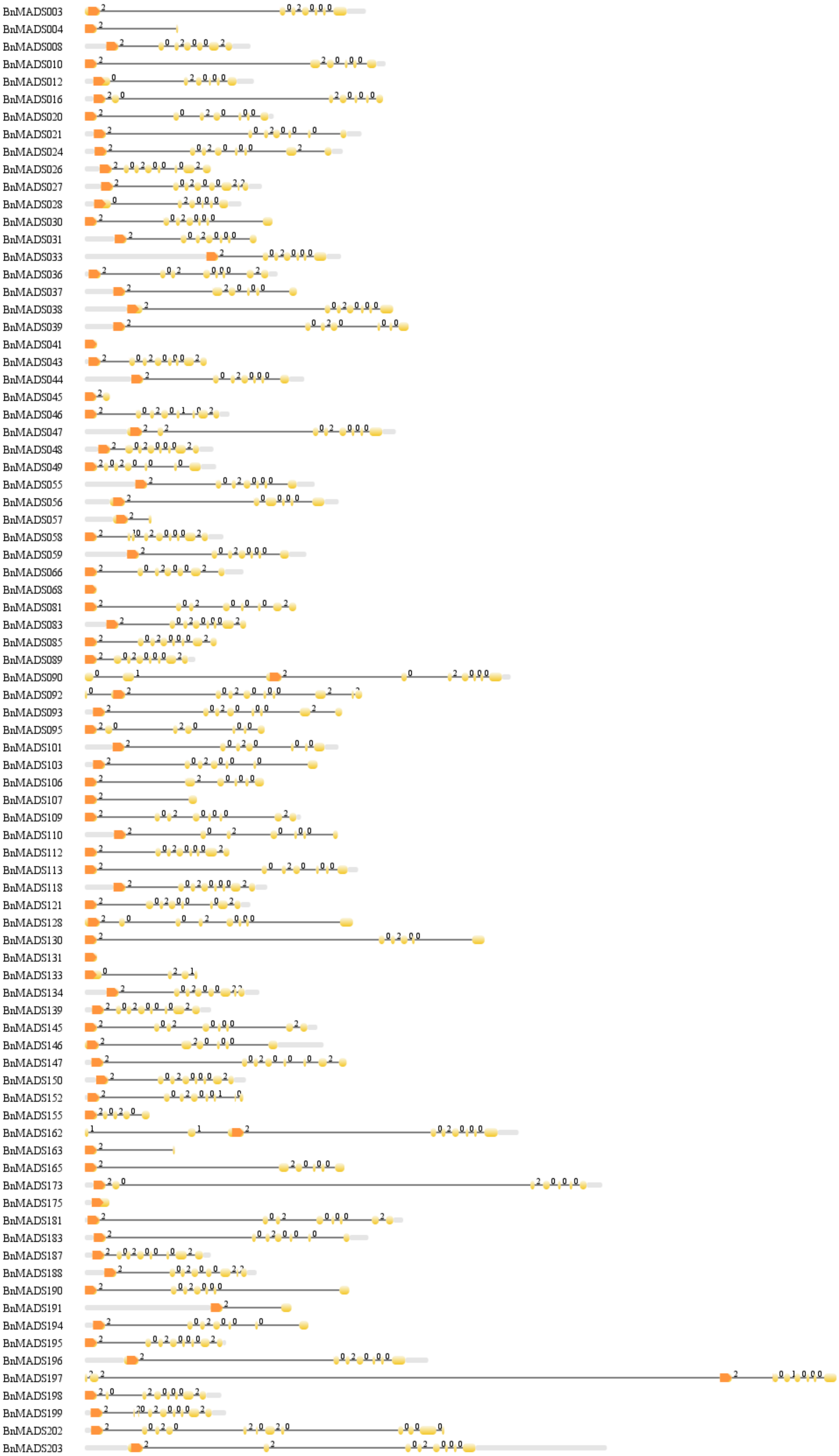
BnMADS003
BnMADS004
BnMADS008
BnMADS010
BnMADS012
BnMADS016
BnMADS020
BnMADS021
BnMADS024
BnMADS026
BnMADS027
BnMADS028
BnMADS030
BnMADS031
BnMADS033
BnMADS036
BnMADS037
BnMADS038
BnMADS039
BnMADS041
BnMADS043
BnMADS044
BnMADS045
BnMADS046
BnMADS047
BnMADS048
BnMADS049
BnMADS055
BnMADS056
BnMADS057
BnMADS058
BnMADS059
BnMADS066
BnMADS068
BnMADS081
BnMADS083
BnMADS085
BnMADS089
BnMADS090
BnMADS092
BnMADS093
BnMADS095
BnMADS101
BnMADS103
BnMADS106
BnMADS107
BnMADS109
BnMADS110
BnMADS112
BnMADS113
BnMADS118
BnMADS121
BnMADS128
BnMADS130
BnMADS131
BnMADS133
BnMADS134
BnMADS139
BnMADS145
BnMADS146
BnMADS147
BnMADS150
BnMADS152
BnMADS155
BnMADS162
BnMADS163
BnMADS165
BnMADS173
BnMADS175
BnMADS181
BnMADS183
BnMADS187
BnMADS188
BnMADS190
BnMADS191
BnMADS194
BnMADS195
BnMADS196
BnMADS197
BnMADS198
BnMADS199
BnMADS202
BnMADS203

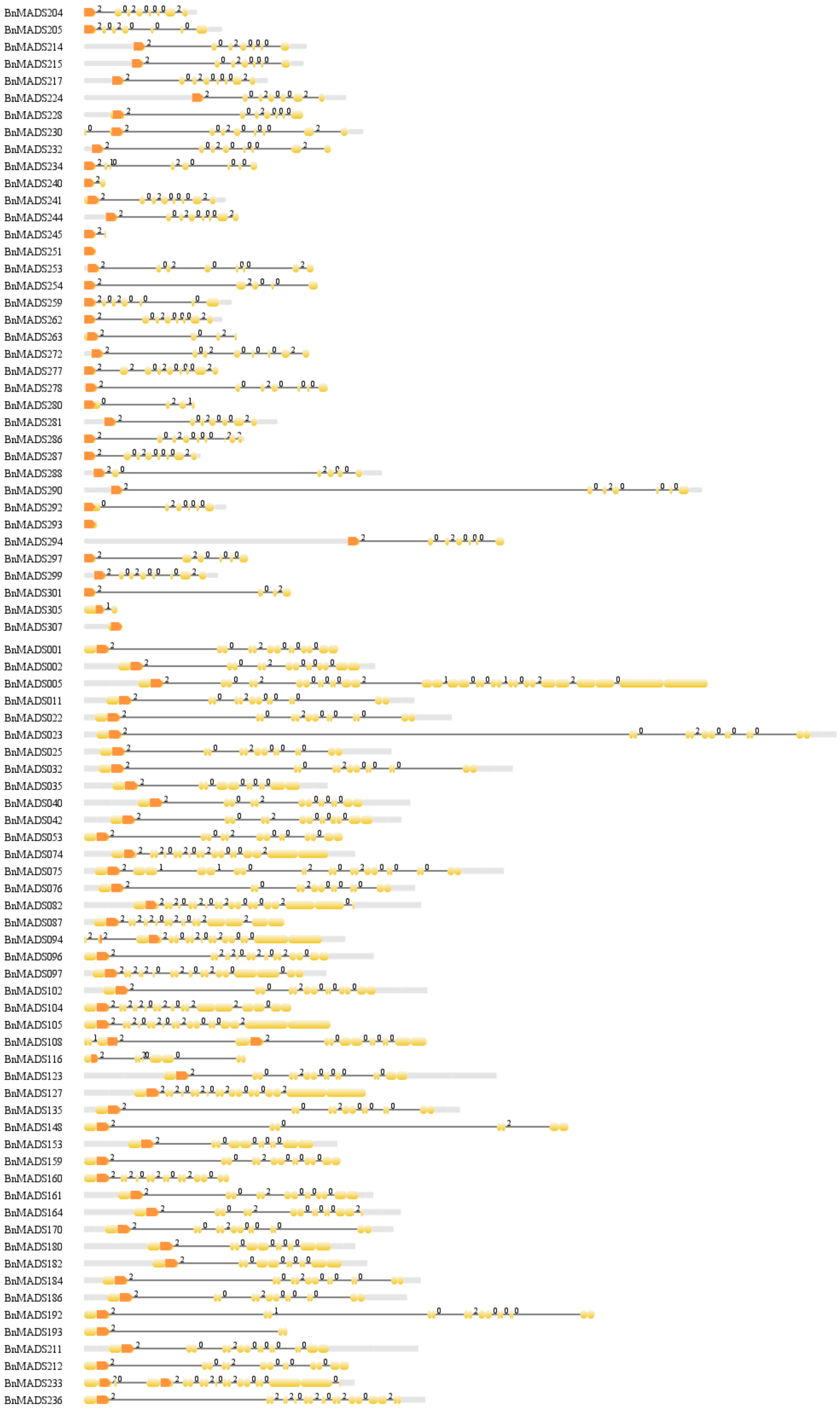
BnMADS204
BnMADS205
BnMADS214
BnMADS215
BnMADS217
BnMADS224
BnMADS228
BnMADS230
BnMADS232
BnMADS234
BnMADS240
BnMADS241
BnMADS244
BnMADS245
BnMADS251
BnMADS253
BnMADS254
BnMADS259
BnMADS262
BnMADS263
BnMADS272
BnMADS277
BnMADS278
BnMADS280
BnMADS281
BnMADS286
BnMADS287
BnMADS288
BnMADS290
BnMADS292
BnMADS293
BnMADS294
BnMADS297
BnMADS299
BnMADS301
BnMADS305
BnMADS307
BnMADS001
BnMADS002
BnMADS005
BnMADS011
BnMADS022
BnMADS023
BnMADS025
BnMADS032
BnMADS035
BnMADS040
BnMADS042
BnMADS053
BnMADS074
BnMADS075
BnMADS076
BnMADS082
BnMADS087
BnMADS094
BnMADS096
BnMADS097
BnMADS102
BnMADS104
BnMADS105
BnMADS108
BnMADS116
BnMADS123
BnMADS127
BnMADS135
BnMADS148
BnMADS153
BnMADS159
BnMADS160
BnMADS161
BnMADS164
BnMADS170
BnMADS180
BnMADS182
BnMADS184
BnMADS186
BnMADS192
BnMADS193
BnMADS211
BnMADS212
BnMADS233
BnMADS236

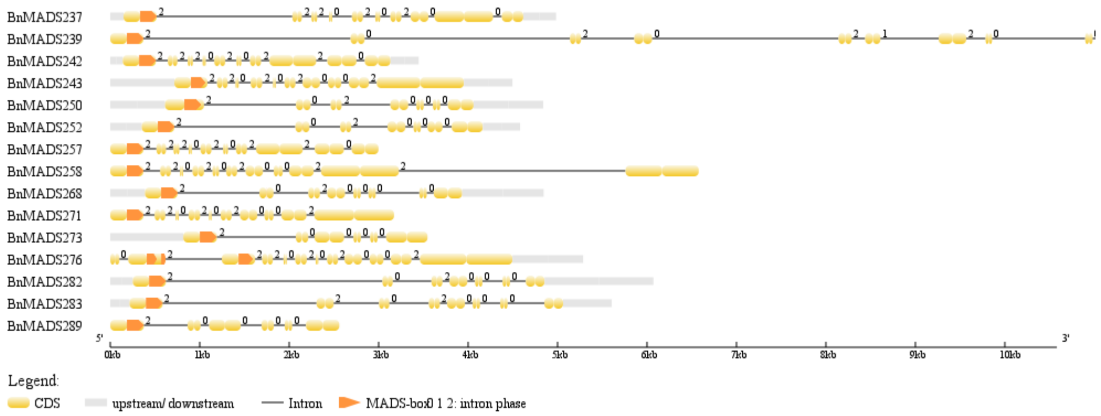

(B)

S2 Fig. Structures of the *BnMADSs* in *B. napus*. Exon(s) are indicated by yellow boxes, MADS-box domain(s) by orange boxes, untranslated region(s) by grey boxes, and spaces between the coloured boxes correspond to introns. Exon and intron size can be estimated using the horizontal scale bar. (A): the structures of type I *BnMADSs*; (B): the structures of type II *BnMADSs*.
